# Ruptured Rudimentary Horn Pregnancy Revealed on Emergency Laparotomy: A Case of Primigravida Presenting in a Developing Country

**DOI:** 10.7759/cureus.2591

**Published:** 2018-05-08

**Authors:** Afshan Hussain, Hafsa Jawaid, Nida Faisal, Naima Shah, Nusrat S Kamal

**Affiliations:** 1 Dow Medical College and Civil Hospital Karachi, Karachi, PAK; 2 Department of Obstetrics and Gynecology, Dow Medical College and Civil Hospital Karachi, Karachi, PAK

**Keywords:** obstetrics, rudimentary horn pregnancy, non communicating horn, unicornuate uterus, emergency exploratory laparotomies, ruptured uterus, mullerian duct, uterine development, primigravida, developing countries

## Abstract

Developmental defects in the Mullerian ducts can result in several uterine anomalies including a unicornuate uterus with a rudimentary horn. Pregnancy in a rudimentary horn is extremely rare occurring in 1:76,000-1:160,000 pregnancies. We present a case of an 18-year-old primigravida with a ruptured rudimentary horn pregnancy (RHP) presenting as acute abdomen at 17 weeks of gestation. Hemodynamic instability led us to perform a life-saving emergency laparotomy. A 1.5 liters hemoperitoneum was encountered with a ruptured non-communicating rudimentary horn. Excision of the horn and unilateral salpingectomy was performed. In addition to ectopic pregnancy and appendicitis, rare uterine anomalies must also be considered in patients presenting with an acute abdomen especially after 10 weeks of gestation. Basic diagnostic facilities may not be available in developing countries. Therefore, clinicians must be aware of this rare entity in order to manage such patients efficiently.

## Introduction

Several uterine anomalies occur due to developmental defects in the Mullerian duct, the incidence of which ranges from 0.1%-3% [1]. Unilateral hypoplasia of the ducts result in a unicornuate uterus with a rudimentary horn. A rudimentary horn pregnancy (RHP) may occur through transperitoneal migration of spermatozoa or fertilized ovum followed by implantation in the horn [1]. Such pregnancies occur in 1:76,000-1:160,000 cases, making it a rare obstetrical condition [2].

## Case presentation

**Figure 1:**
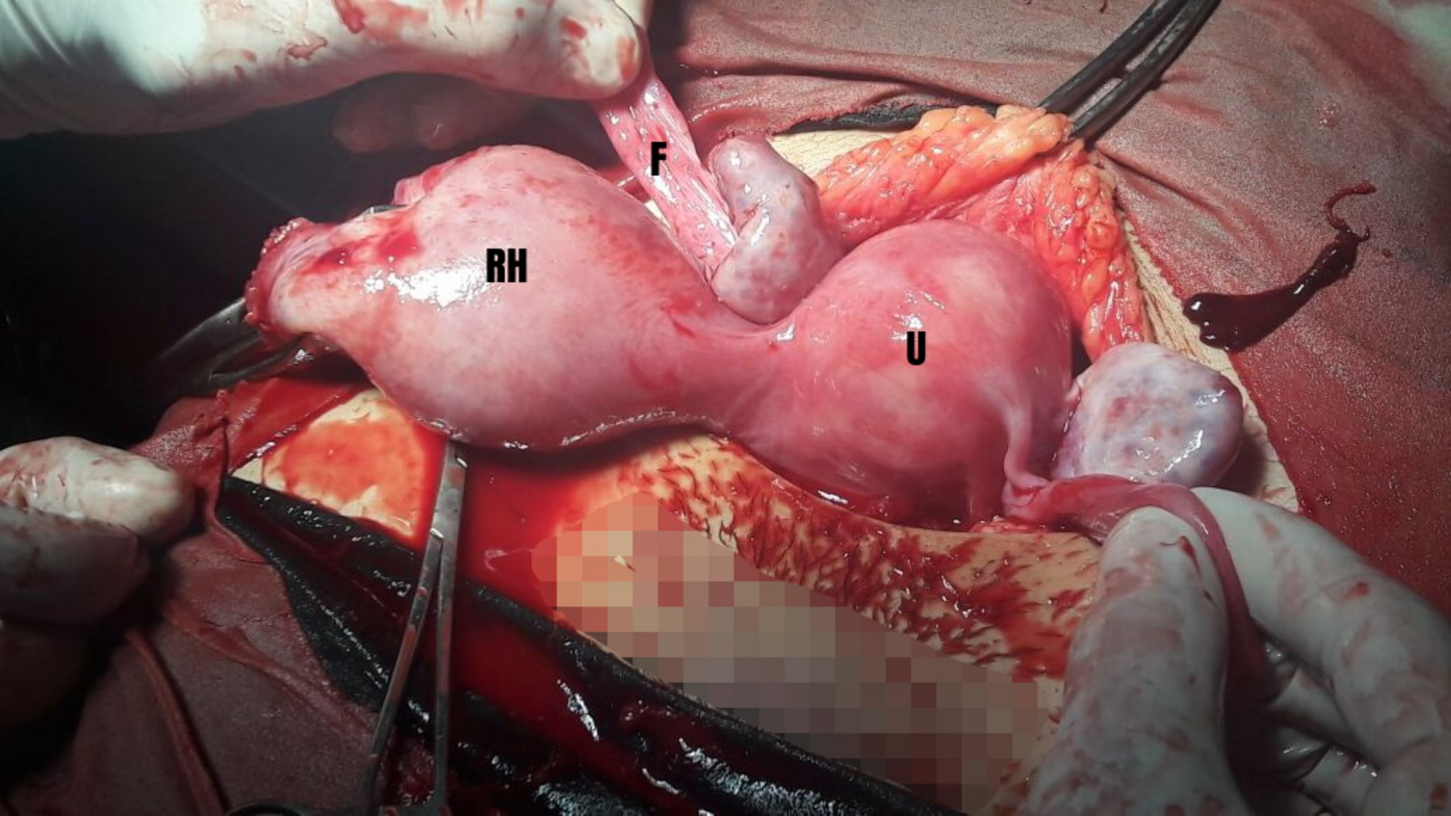
Non-communicating rudimentary horn (RH) and its fallopian tube (F). (U) represents uterus.

**Figure 2:**
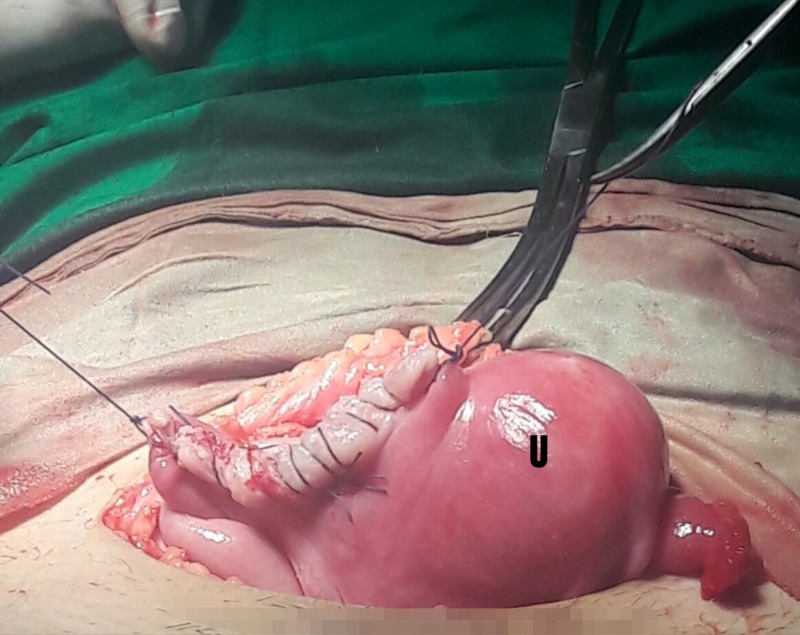
Uterus (U) post non-communicating rudimentary horn excision.

An 18-year-old primigravida, at 17^th^ week of gestation by last menstrual period, presented to the emergency department with complaints of severe abdominal pain and two episodes of syncope since last three hours. The pregnancy followed spontaneous conception in a non-consanguineous marriage of six months and was confirmed by a urine pregnancy test at home. She developed acute, severe, lower abdominal pain, stabbing in nature and associated with shoulder tip pain. There was no history of dysmenorrhea prior to pregnancy. Past medical and surgical history was insignificant. She was an unbooked case, hence no records of routine pregnancy scans or investigations were available. On arrival, the patient was conscious, well-oriented, and afebrile. General examination revealed severe pallor and hypovolemic shock. Blood pressure was 50/30 mmHg and pulse rate was 120 beats per minute. The abdomen was found to be tense, tender, and mildly distended. Pelvic examination demonstrated a healthy vagina. The cervical os was closed with no evidence of bleeding. The size of the uterus could not be determined due to intense guarding. Cervical excitation was found to be positive. Tenderness and fullness were present in both fornices. Her investigations showed hemoglobin of 7.6 g/dl and raised total leukocyte count of 22.5 x10^3^ while serum electrolytes, urea, creatinine, and coagulation profiles were normal. She was immediately managed with two intravenous cannulas for fluid resuscitation and was catheterized. Blood was sent for cross match and four units of blood were arranged. A provisional diagnosis of ectopic pregnancy was made and she was transferred to the operation theatre for emergency exploratory laparotomy. The abdomen was opened by a Pfannenstiel incision and a hemoperitoneum of 1.5 liters was encountered along with an extrauterine 17-week fetus floating in the peritoneal cavity. Further exploration revealed a right-sided ruptured non-communicating rudimentary horn (5 cm x 4 cm) attached to the uterine cavity with a 2.5 cm fibrous band (Figure 1). Both the ovaries and left Fallopian tube were found to be healthy. The rudimentary horn was excised and a right salpingectomy was performed and hemostasis was secured. The patient received two packs of blood intraoperatively. The resected uterine segment was repaired (Figure 2) followed by closure of the abdomen.

Intravenous urography was performed which revealed no urological abnormalities. Histopathologic examination of the specimen confirmed the diagnosis of a unicornuate uterus with a non-communicating rudimentary horn. Following an uncomplicated recovery, the patient was discharged on the fourth postoperative day.

## Discussion

Pakistan has had limited success in reducing maternal and neonatal deaths with a maternal mortality ratio of 276 per 100,000 pregnancies [3]. Previous prospective community-based surveys of the Global Network concluded lack of maternal education along with insufficient antenatal and neonatal care interventions to be strongly indicative of poor maternal outcome in the country [4]. We report the case of a primigravida in a developing country who presented with a rare condition discovered during surgery without any prior imaging to facilitate the diagnosis. There was no portable ultrasound available at the facility site or skilled staff to manage the transfer at the time of presentation without any delay in management. Lack of previous scans and available services led us to manage the patient solely based on her history, clinical examination, and lab investigations and execute a life-saving emergency laparotomy. Although there has been a case of normal vaginal delivery reported in rural Nigeria [5], most cases of RHP result in uterine rupture and seldom cases progress to full term pregnancy. A high maternal and fetal mortality is associated with RHP, and thus, a timely diagnosis is vital to save the life of the patient [1]. According to Shah and Khan [6], ectopic pregnancy should be suspected in every pregnant lady presenting with unidentified pain unless proven otherwise. Hence, in our case, differentials fluctuated between acute appendicitis and ruptured ectopic pregnancy. However, it is important to note that ectopic pregnancies present commonly between the sixth and tenth weeks of gestation, whereas a RHP may present late [7]. Therefore, rare causes of acute abdomen during pregnancy such as RHP must be considered in a patient presenting after 10 weeks of gestation. Most cases of RHP are misdiagnosed through ultrasound as tubal, cornual, intrauterine, and abdominal pregnancy [2]. The rate of misdiagnosis is 74% [1], and it is missed even by the most experienced radiologist probably due to its rarity and non-familiarity with this potentially lethal condition. In contrast, magnetic resonance imaging (MRI) is a useful, non-invasive diagnostic tool with higher accuracy for diagnosis of Mullerian duct abnormalities. However, it is difficult to perform in acute presentations requiring immediate intervention [8]. RHP is best managed surgically with excision of the uterine horn which combats high risk of recurrence. MRI can help outline the anatomical relation between the uterus and the rudimentary horn. Both can be connected either by a band of tissue making transection easier, or via a thick firm attachment associated with higher risks of dissection into the uterine cavity [9]. A strong association of urinary tract anomalies with unicornuate uterus is present, making it vital for the patient to be thoroughly examined for urological problems [8]. Hence, an intravenous pyelogram was performed postoperatively which ruled out urological anomalies.

## Conclusions

The diagnosis of RHP is particularly difficult in developing countries such as Pakistan. Substandard antenatal care and lack of access to basic diagnostic services such as ultrasonography and MRI may cause delayed recognition and management of several clinical anomalies contributing to maternal and perinatal mortality. Therefore, clinicians must be aware of this rare entity in order to achieve timely management.
